# Flexible and configurable embedded electrical energy measurement system to acquire and process high-frequency features

**DOI:** 10.1016/j.ohx.2024.e00539

**Published:** 2024-05-31

**Authors:** Maximiliano E. Véliz, Gustavo E. Real, Alejandro D. Otero

**Affiliations:** a Universidad Nacional de Gral. Sarmiento, Instituto de Industria, Juan María Gutiérrez, 1150, Los Polvorines, Provincia de Buenos Aires, Argentina; b Universidad Nacional de Hurlingham, Secretaría de Investigación, Av. Gdor Vergara, 2222, Ciudad de Tesei, Provincia de Buenos Aires, Argentina; c Universidad de Buenos Aires, Facultad de Ingeniería, Departamento de Energía, Paseo Colón 850, 1063, Buenos Aires, Argentina; d CONICET. Centro de Simulación Computacional para Aplicaciones Tecnológicas, Godoy Cruz 2390, 1425, CABA, Buenos Aires, Argentina

**Keywords:** Energy disaggregation, High-frequency signal, Embedded system

## Abstract

A novel High-Frequency Electric Energy Metering System to inspect non-conventional features that may be relevant for studying real-time energy disaggregation and control of household appliances is presented. Integration of a data acquisition and control board, designed and built to be assembled with an Arduino Due, with the M90E36A Demo Board, allows for flexible and configurable electrical energy measurements. A key feature is that up to 4 current channels can be measured synchronously. On the one hand, samples can be obtained and processed by the M90E36A IC internal Digital Signal Processor at 3 Hz in the time domain and 2 Hz in the frequency domain. On the other hand, the M90E36A IC direct access memory mode can be operated, allowing 8 kHz pure voltage and current signals to be obtained. Finally, integration with Raspberry Pi allows to design and incorporate a custom signal processor into the study. Additionally, in this article, an application example is presented where the variation of the residual harmonic components of a household appliance is obtained.

## Graphical abstract

**Figure:**
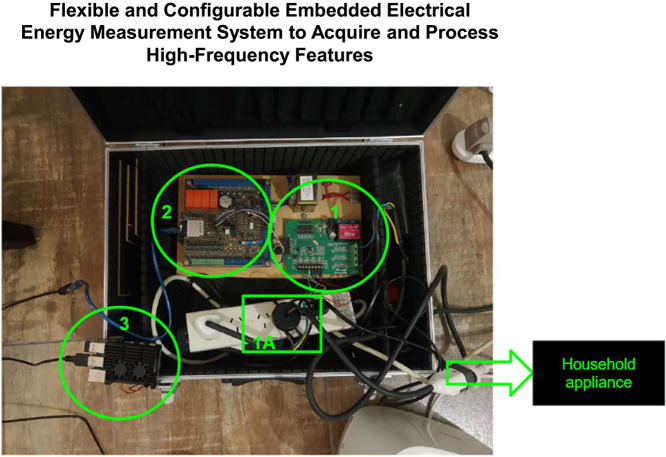

## Specifications table

**Table:** 

**Hardware name**	The Arcane Meter

**Subject area**	•*Engineering and material science*

**Hardware type**	•*Electrical engineering and computer science*

**Closest commercial analog**	•*Power Quality Analyzer UT285C* (https://meters.uni-trend.com/product/ut285c/). • *Dranetz PowerGuiaTM 440S* (https://www.dranetz.com/wpcontent/uploads/2014/02/440S-UsersGuide-RevC.pdf).

**Open source license**	*Creative International Commons Attribution 4.0*

**Cost of hardware**	*$ 1133*

**Source file repository**	https://doi.org/10.5281/zenodo.7802260

## 1 Hardware in context

In the context of Non-Introduction Load Monitoring (NILM), researchers need access to data from appliance and household consumption to develop, test, and evaluate energy consumption breakdown algorithms. The REDD (Reference Energy Disaggregation Dataset) was published in 2011 [1]. It is the first publicly available dataset exclusively designed to study non-invasive load measurement algorithms. Latter, other publicly available datasets have emerged, each with its own sampling characteristics. Examples include UK-Dale [2], AMPD [3], AMPDs2 [4], ECO [5], REFIT [6] and GREEND [7]. Comparing the available data sets [6], [8], [9], it becomes clear that there is no universally accepted criterion for creating a database to study non-invasive load monitoring. One of the reasons for this is that, according to the approach adopted, the type of data presented may change significantly depending on the macroscopic or microscopic properties of the loads studied, the frequency of the load signal sampling, etc. [10].

A survey of the energy meters used to create those datasets, reveals that in most cases commercial products were used. That is, energy meters whose design features are not available to the end user of the product and where there is no access to the firmware in order to create a configuration other than the factory settings. This means that the internal test frequency of the device cannot be controlled and information about the measured magnitudes is only available after transmission. In general, the data reporting rate has lower resolution than the test frequency, with some internal processing in between. The work of [11] remarks the need to provide access to the firmware to enable new applications of smart meters. There is a variety of sampling frequencies adopted both for the individual appliances, which range from 1 Hz to 1/6 Hz, and the aggregated current, reaching up to 16 kHz. Some datasets even report at different frequencies in both cases, and those reporting at high frequencies present either unprocessed signals or signals processed by standard techniques.

Typical professional electrical energy analyzers have proprietary firmware and integrated Digital Signal Processors (DSP) based on conventional functions that report electrical magnitudes in Root Mean Square (RMS) form in the time domain, such as current, voltage, power, and the amplitude of the harmonic components in the frequency domain. Integration with other hardware is not possible without access to the proprietary firmware, and generally there is no access to pure sampling outside the integrated DSP, which means that one is limited to obtaining conventional electrical power and quality parameters.

In this context, devising an embedded system capable of reporting raw quantities at high frequency appears as a fundamental step in the development of both new disaggregation techniques and future monitoring hardware. Moreover, the design of *ad hoc* hardware [12] with controllable monitoring configurations seems an interesting possibility, even more so if several devices can be monitored in a synchronized manner by a single instrument.

To this end, we present the design and implementation of an embedded system incorporating the Atmel M90E36A IC, which has its own public firmware, allowing either feature extraction via the integrated DSP, or obtaining pure samples via Direct Memory Access (DMA) mode, for subsequent processing in user-developed applications. Previous hardware development using the Atmel M90E36A IC [13] and Teensy® USB Development Board for high-frequency analysis of load features can be found, for example in [14], with a focus on the aggregated signal processing but not on the synchronous measurements and customizable DSP.

## 2 Hardware description

Compared to the usual commercial power analyzers, the presented embedded system:

• offers a flexible open-source solution which integrates components with open specifications and programmable firmwares,
• gives conventionally processed samples with sampling rates of up to 3 Hz in the time domain and 2 Hz in the frequency domain,
• could provide pure samples (hexadecimal string of the M90E36A IC) at 8 kHz for further processing in a custom DSP hosted on the Raspberry Pi,
• allows for simultaneous sensing of up to four independent signals, a typical set up being 1 aggregated signal and 3 individual appliances,
• data extraction can be done from a free serial port terminal which allows to integrate the system acquisition with the data processing (in this case, in the Raspberry Pi) in a direct way. Commercial energy meters, usually need a proprietary software for data extraction and there is no easy procedure to be integrated with other softwares.

**Fig. 1:**
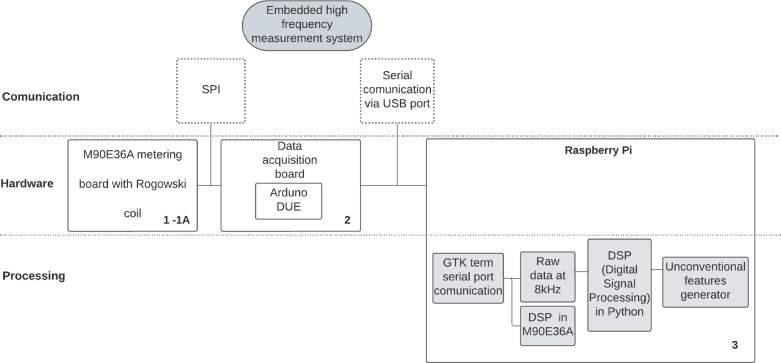
Embedded system block diagram [12].

Fig. 1 shows the block diagram of the measuring system. Hardware integration includes the M90E36A-Demo Board (DB) (#1), which communicates through the Serial Peripheral Interface (SPI) with the acquisition and control board (#2) which includes an Arduino Due. The software developed in Arduino IDE provides the firmware and menu operations to access the features of the M90E36A IC. Using a serial console port (hosted on the Raspberry Pi (#3)), it is possible to enter the user menu and acquire processed samples from the Atmel DSP M90E36A IC at a frequency of 3 Hz in the time domain and 2 Hz in the frequency domain. It is also possible to acquire pure samples (hexa strings) to be processed by *user-defined* DSPs on the Raspberry Pi. The Raspberry Pi acts as the user interface, collecting and processing data and interacting with the rest of the system via the serial bus. The idea is to create a compact standalone system, but an external PC could be used instead.

**Fig. 2:**
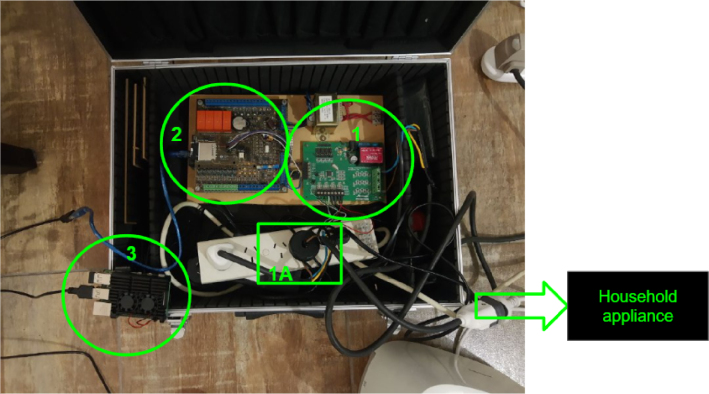
High-frequency measurement system implementation.

Fig. 2 shows the actual assembly of the measurement system. The hardware components that appear in the figure are:

• **1A:** Rogowski coil PA3202NL (×4)
• **1:** M90E36A-DB (×1)
• **2:** Data acquisition and control board (×1) ＋ Arduino Due microcontroller board (×1)
• **3:** Raspberry Pi 4 (×1)

The M90E36A-DB (#1) incorporates 7 independent 2nd order sigma-delta Analog to Digital Converter (ADC), which could be employed in three voltage channels (phase A, B and C) and four current channels (phase A, B, C, and neutral line) in a typical three-phase four-wire system. It is used for the demo and testing of the M90E36A IC. In the application note [15], the M90E36A IC is defined as a poly-phase high performance wide-dynamic range metering IC. The most important feature we valued when designing the embedded system is that the M90E36A IC supports a SPI master mode, called DMA mode. In this mode, the M90E36A ADC sends raw data to the external Micro controller Unit (MCU) at a speed of up to 1800kbps and the ADC samples (at 8K sample rate) continuously.

The Rogowski coil (#1A) measurement method measures the current derivative, which prevents its usage in measuring DC currents. The Rogowski coil has some significant features that have attracted attention in recent years. Some of its main advantages are [16]:

• Bearing large overloads without damage;
• Measuring currents in an extensive range, without saturation;
• Non-intrusive nature (drawing no power form the main circuit);
• Wide bandwidth, in a range of 0.1 Hz to 1 GHz;
• Excellent transient response;
• Safety (electrically isolated from the main circuit);
• Linearity in the entire working range.

**Fig. 3:**
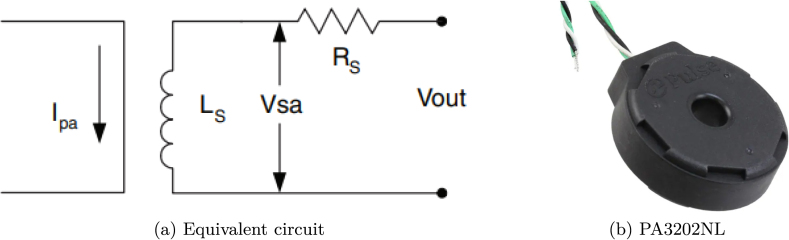
Rogowski coil. Data sheet [17].

Considering the application to high-frequency current and voltage measurement in a transient synchronous regime of up to three household appliances and the aggregate signal, it was decided to use the Rogowski coil PA3202NL shown in Fig. 3, where $L_{s}$ is the secondary winding inductance; $R_{s}$, the secondary winding resistance; $V_{out}$, the secondary voltage. Given $I_{pa}$, the actual primary current; $k_{r}$, the rated transformation constant, and $F_{r}$, the frequency sinusoidal waveforms, the actual secondary output voltage $V_{sa}$, results:

(1) $$V_{sa}=k_{r}\;F_{r}\;I_{pa}.$$

The PA3202NL model has 0.2 accuracy class as per IEC 60044-1. In this case, $V_{sa}=416\;\mu \mathrm{V/A}$, $R_{s}=53.7\;\Omega$ and $k_{r}=8.33\;\mu \Omega /\mathrm{Hz}$ [17]. Although the 0.2 accuracy class is not particularly important for NILM application research, the recommendations of the M90E36A application note [15] were followed. Given the low cost of these coils, the objective of a low cost embedded system was not compromised.

The acquisition and control board (#2) provides access to an SD memory reader and a real-time clock, in addition to having additional analog inputs and outputs that could be used for control loops. Furthermore, regarding communications, the acquisition board provides RS-485, RS-232, and CAN communication buses for specific applications that could require appliance on-off control. The acquisition and control board has the following input/output features:

• 8 opto-coupled digital inputs - 0 to 24 V;
• 8 analog inputs (single ended) - 0 to 10 V, 4 to 20 mA, selectable with jumpers;
• 4 instrumentation analog inputs for special measurements 0 to 3.3 V to 0.5 mA, software programmable gain.
• four relay outputs - 24 V @ 1 A.
• four open drain outputs - 24 V @ 0.5 A.
• four PWM outputs - 0 to 3.3 V @ 10 mA.
• two analog outputs - 0 to 10 V, 0 to 20 mA, jumper selectable.

For the specific purposes of the application of feature acquisition on household appliances for the non-intrusive load monitoring study, the analog instrumentation channels of the ACQII are not used.

## 3 Design files summary

**Table:** 

Design filename	File type	Open source license	Location of the file
**P1**: arduino_main_program.ino	*software (Arduino IDE)*	*Creative Commons Attribution 4.0 International*	https://doi.org/10.5281/zenodo.7802260

**P2**: DMA_samples _convertion.ipynb	*software(Python notebook)*	*Creative Commons Attribution 4.0 International*	https://doi.org/10.5281/zenodo.7802260

**P3**: library_ descriptor_M90E36A.h	*software (Arduino IDE)*	*Creative Commons Attribution 4.0 International*	https://doi.org/10.5281/zenodo.7802260

**P4**: acquisition_control _board_schematic.pdf	*PDF*	*Creative Commons Attribution 4.0 International*	https://doi.org/10.5281/zenodo.7802260

**P5**: acquisition_ control_board_TopView.jpg	*JPG*	*Creative Commons Attribution 4.0 International*	https://doi.org/10.5281/zenodo.7802260

**P6**: acquisition_control _board_BottomView.jpg	*JPG*	*Creative Commons Attribution 4.0 International*	https://doi.org/10.5281/zenodo.7802260

**P7**: acquisition_control _board_CADCAM.rar	*plain text files. Software (PROTEUS)*	*Creative Commons Attribution 4.0 International*	https://doi.org/10.5281/zenodo.7802260

For each design file listed in the summary table above, include a short description of the file below (just one or two sentences per design file).

• **P1**: arduino_main_program.ino[18]
  Main operating program of the embedded system. The software developed in Arduino IDE contains the firmware of the embedded system, the M90E36A IC registers, the operating and communications functions for the Arduino Due, and the user interface for the operation of the equipment. It includes the necessary libraries, input and output settings, CAN communication definitions, state machine definitions, software reset definitions, SPI bus settings, operating routines, subroutines, etc.
• **P2**: DMA_samples_convertion.ipynb[18]
  Python code that contains the application function to transform the direct pure hexadecimal samples of the M90E36A IC into decimal samples. This code is used when the embedded system is working in DMA mode. Receive hexa-strings (.txt file) to process.
• **P3**:library_descriptor_M90E36A.h[18]
  Library created for the Arduino board. It has the pin and registers definitions for Microchip/Atmel M90E36A IC. Important registers include low-power mode registers, calibration registers, measurement calibration, and fundamental/harmonic energy calibration registers, among others.
• **P4**:acquisition_control_board_schematic.pdf
  Schematic design for the data acquisition and control board.
• **P5**:acquisition_control_board_TopView.jpg
  Figure of the finished board top view.
• **P6**:acquisition_control_board_BottomView.jpg
  Figure of the finished board bottom view.
• **P7**:acquisition_control_board_CADCAM.rar
  CAD–CAM files for the acquisition and control board construction. Include Photoplotter setup and specifications in plain text files:
  1. acquisition_control_board_CADCAM_Readme.txt
  2. acquisition_control_board_CADCAM_Top_Copper.txt
  3. acquisition_control_board_CADCAM_Inner_1.txt
  4. acquisition_control_board_CADCAM_Inner_2.txt
  5. acquisition_control_board_CADCAM_Bottom_Copper.txt
  6. acquisition_control_board_CADCAM_Top_Silk_Screen.txt
  7. acquisition_control_board_CADCAM_Bottom_Silk_Screen.txt
  8. acquisition_control_board_CADCAM_Top_Solder_Resist.txt
  9. acquisition_control_board_CADCAM_Bottom_Solder_Resist.txt
  10. acquisition_control_board_CADCAM_Top_SMT_Paste_Mask.txt
  11. acquisition_control_board_CADCAM_Bottom_SMT_Paste_Mask.txt
  12. acquisition_control_board_CADCAM_Mechanical_1.txt
  13. acquisition_control_board_CADCAM_Drill.txt

## 4 Bill of materials summary

The full version of the Bill of Materials that contains electronic component details is available with the supplementary material and the list of commands (user_command_list.txt) for the user menu [19].

## 5 Build instructions

### 5.1 Board manufacture

**Fig. 4:**
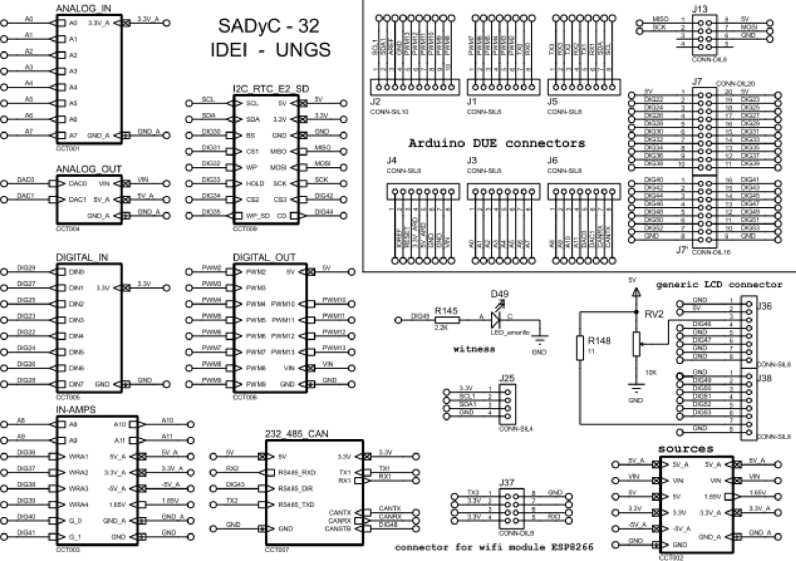
Acquisition and control board components.

**Fig. 5:**
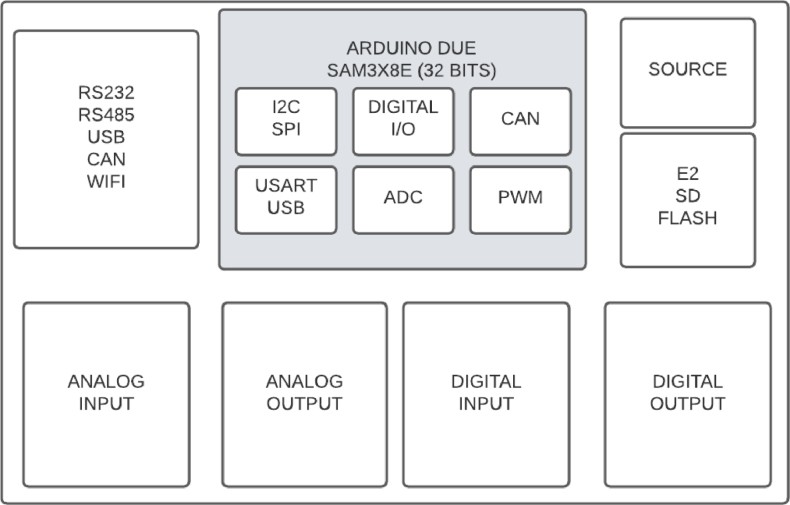
Data acquisition board block diagram [20].

Manufacture the acquisition and control board using part list **P4** and **P7** file found in [19]. The main components are shown in Fig. 4 and the block diagram in Fig. 5.

### 5.2 Assemblage

**STEP 1:** Data acquisition and control board ＋ Arduino Due (this assembly is called ACQII from now on).

**Fig. 6:**
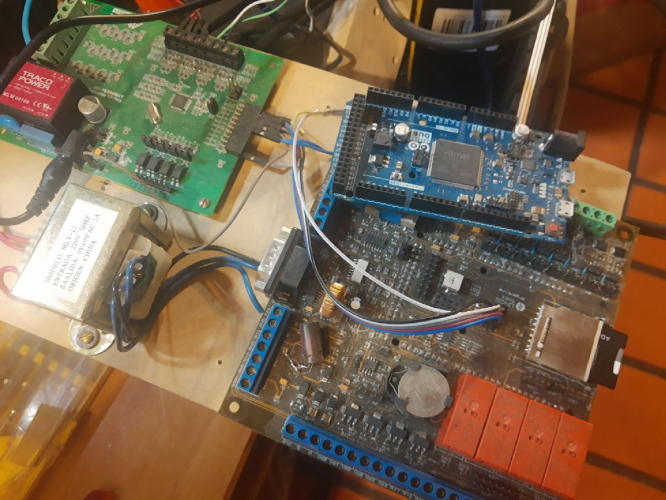
Assembly of Arduino Due within the acquisition and control board.

The Arduino Due board is inserted in the data acquisition and control board [20] into the corresponding socket (see Fig. 6) to provide access to an SD memory reader and a real-time clock, in addition to having additional analog inputs and outputs that could be used for control loops. The components of the data acquisition board are shown in Fig. 4.

**STEP 2:** ACQII ＋ M90E36A-DB.

**Fig. 7:**
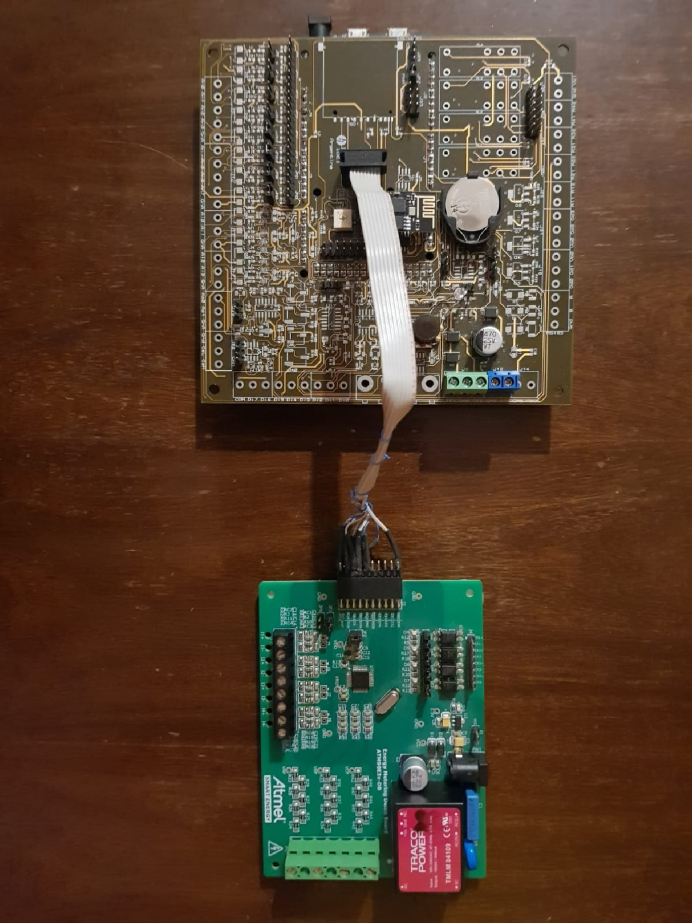
Communicating ACQII board with M90E36A-DB.

**Fig. 8:**
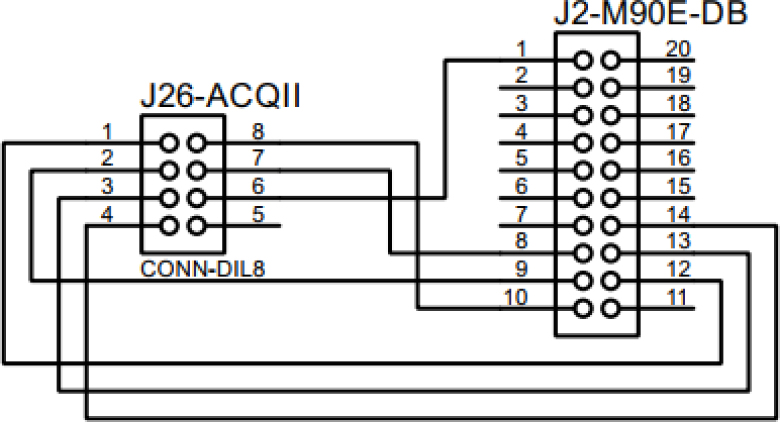
ACQII ＋ M90E36A-DB connection schematic.

The next step is to communicate ACQII with the M90E32A-DB. The connection between devices is directly affected by the type of communication chosen. For the communication between ACQII module and the M90E36A IC we use the SPI protocol, which makes 4 connections called SDI, SDO, SCLK and CS. The reason for using the SPI protocol is that the M90E36A chip has this unique way of communication, and the Arduino Due board also supports it. The power supply of the M90E36A-DB was connected to the ACQII outputs. They have a voltage of 3.3 V, the same as the chip. Fig. 7, Fig. 8 show the connection between boards.

**STEP 3:** ACQII ＋ M90E36A-DB ＋ Rogowski Coil PA3202NL.

**Fig. 9:**
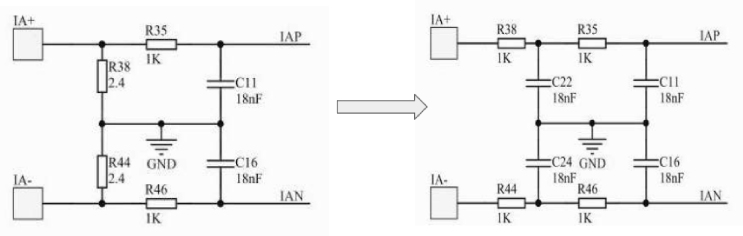
Current filter modification.

The M90E36A-DB supports two types of current transducers: current transformer or Rogowski coil. Each of these requires different types of filters and the printed circuit board is already designed to solder one type of filter or the other. To work with the Rogowski coil it was necessary to change the current input filter of the Atmel measurement board, as it is recommended to use a two-order filter [15]. The implementation is shown in Fig. 9 with the addition of two reactive components (C22 and C24, multilayer chip capacitors) and the modification of two resistors (R38 and R44). This allows more flexibility in the adjustment the frequency response.

**STEP 4:** ACQII ＋ M90E36A-DB ＋ Rogowski coil ＋ Raspberry Pi.

The Arduino Due (from ACQII) connects to a Raspberry Pi via USB serial port communication to access the commands and the DSP required for data processing, as shown in Fig. 2.

## 6 Operation instructions

### 6.1 Loading the Arduino main program and interaction through the serial port terminal

The firmware for the Arduino Due board was written using the Arduino IDE. It includes boot tests, command usage, log modes, and access to the M90E36A IC configuration. The setup includes selecting the operating mode (Table 1) to obtain samples through the particular DSP developed and hosted on the Raspberry Pi or the DSP embedded in the Atmel IC to sample conventional features.

In order to load the program in the Arduino Due, you will need to start the Arduino IDE on a PC and load the **P1** file. Before compiling, the conditional compilation switches must be chosen according to how the acquisition and control board is connected. In the event that it has an integrated Wi-Fi board, the corresponding ‘define’ must be commented off.

**Table 1:** M90E36A IC Operation Modes.

Operation Mode	Description
DMA mode samples at 8 kHz	Hexa string from M90E36A IC processed in the user-defined DSP inside Raspberry Pi
RMS samples at 3 Hz	Signal processing in Atmel embedded DSP

**Fig. 10:**
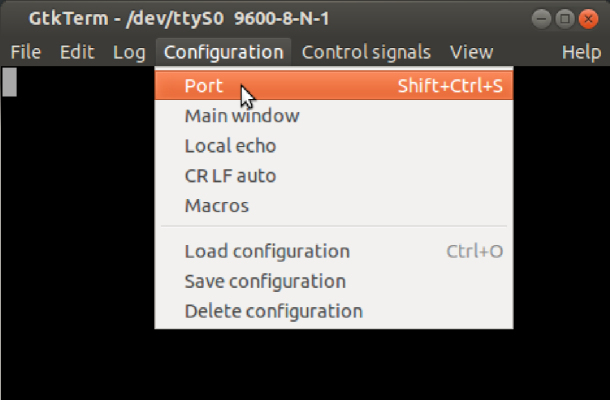
GtkTerm port selection [21].

So, to start working with the system via the console port:

• **STEP 1**: load **P1** file in Arduino IDE from a PC. Modify the ‘define’ you need. Compile and transfer to the Arduino DUE board.
• **STEP 2**: access the Raspberry Pi (operating locally or remotely).
• **STEP 3**: open a serial port terminal (console); in our case, we install and use the GTK-term.^1^
• **STEP 4**: establish communication between the console and Arduino DUE via the corresponding COM port in the terminal port configuration menu (Fig. 10).

The commands to interact with the Atmel IC are transmitted in ASCII format, either through WiFi or console port, according to the format:

 ＋ command^2^ ＋  , where:  = 0x1B (Hexa),  = 0x0D (Hexa).

This way, the original communication format already codified is maintained, adding the possibility of including new commands in a simple manner, just by respecting the corresponding format.

**Fig. 11:**
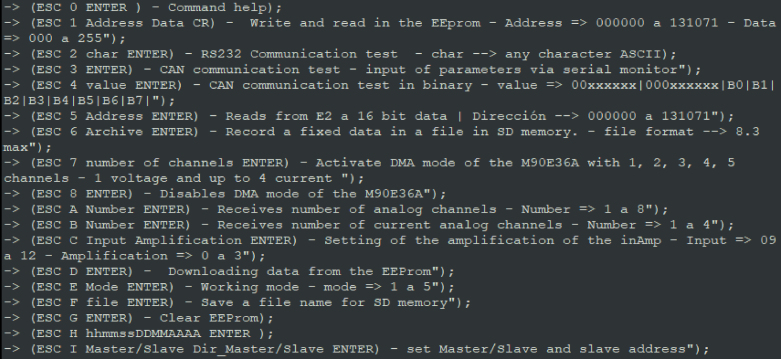
User menu.

• **STEP 5**: in GTK-term, to access the main menu type:  ＋  ＋  (Fig. 11).

### 6.2 DMA mode buffer and pointer operation

**Fig. 12:**
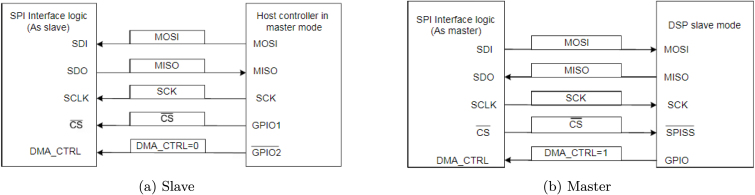
Atmel IC working modes [15].

As mentioned above, communication between the M90E36A-DB and the ACQII is carried out via a serial bus called SPI. It has the advantage of being bidirectional and synchronous. One possible setting is the master–slave configuration (Fig. 12(a)), in which the ACQII MCU performs all polling, and the Atmel IC responds accordingly. The other is the DMA mode (mentioned as master mode in [15]), in which Atmel IC is able to continuously send raw sample data (at a sample rate of 8 kHz) from one board to another without any information exchange protocol until stopped. In the first case, the data is collected and processed in various electronic stages of the Atmel IC (internal DSP). Values for voltage, current, frequency, etc. are obtained at 3 Hz in the time domain and 2 Hz in the frequency domain. However, when for some specific reason pure sampling, i.e. without processing, of the various channels involved is required, the DMA mode is used (Fig. 12(b)). As both signals (voltage and current) needed to be sampled simultaneously for the type of analysis proposed in this work, the master–slave option was no longer feasible and DMA communication in master mode had to be used. The master–slave option was no longer feasible for this specific analysis because in master–slave mode the processed samples are taken from the DSP of the M90E36A. To explore and define new features for non-intrusive load monitoring, the RMS values were not sufficient. Therefore, raw samples must be available for transmission and further processing according to the needs of the project.

The DMA communication mode requires the Atmel IC to work as a master and the Arduino Due board microcontroller to work as a slave. Finally, a Python-implemented program performs the conversion and verification of the data to obtain high-frequency characteristics (see program **P2** as an example). The Python program is used to extract and convert hexadecimal samples from the raw data stream into decimal samples of each channel. It also provides the means to design customized signal processors. In this work, the methodology to obtain the harmonic residual currents is presented in Section 7.2.

**Fig. 13:**
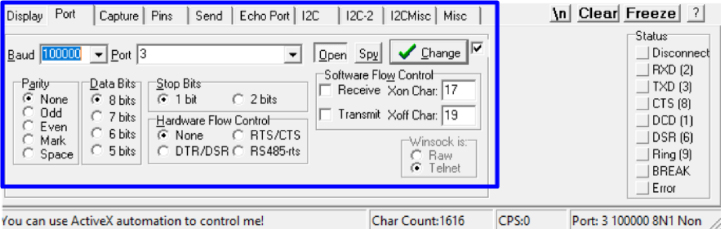
Port opening.

**Fig. 14:**
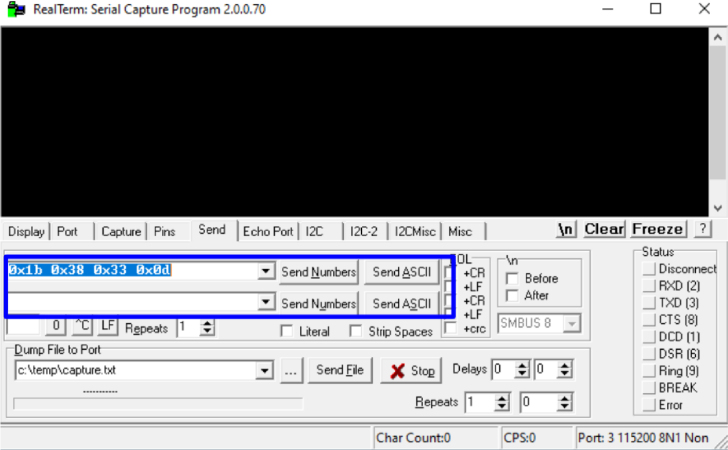
Sending commands.

**Fig. 15:**
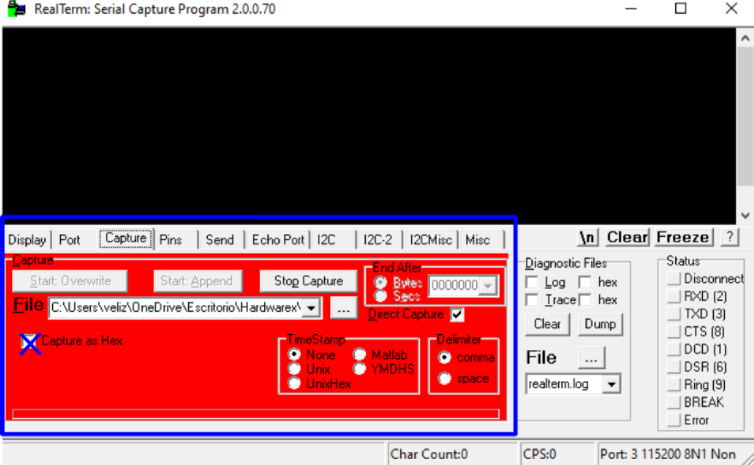
Saving file.

**Fig. 16:**
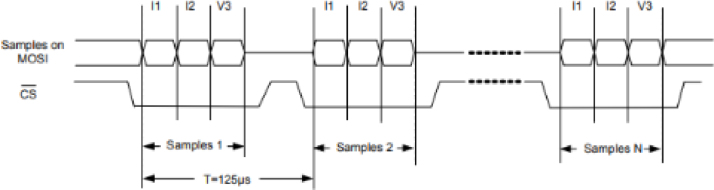
Sample sequence example [13].

**Fig. 17:**
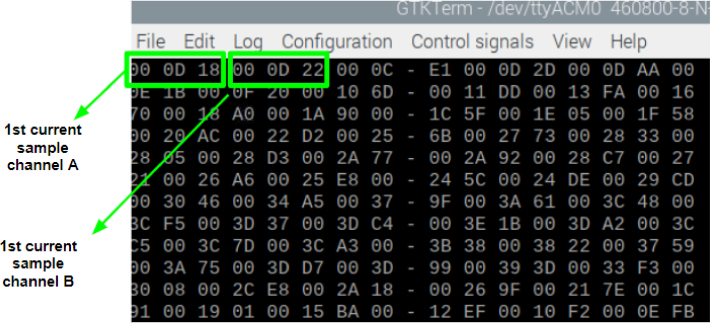
Example of hexa-string captured in console.

**Fig. 18:**
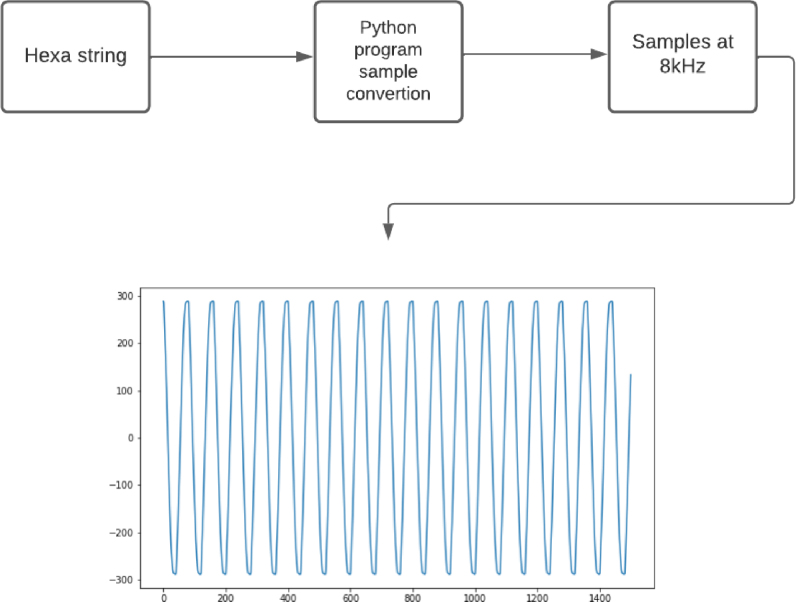
Samples conversion.

**Fig. 19:**
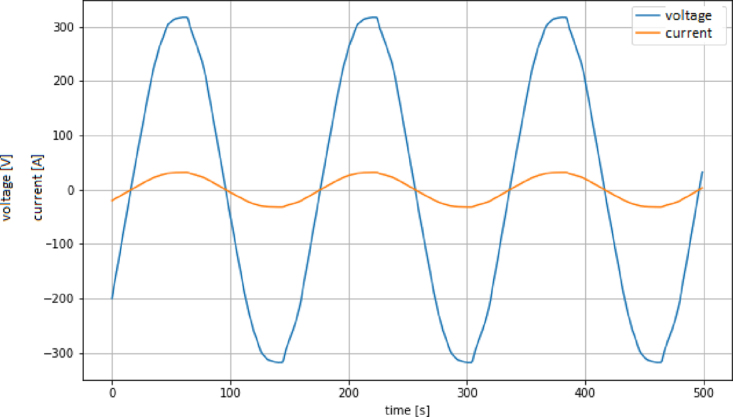
Voltage and current in a toaster, DMA operation.

To access the DMA mode through the console and begin to record the samples of a number of channels:

• Set the transmission speed of the console to 100000 bauds^3^ in port configuration menu (Fig. 10). The maximum sampling rate supported by the M90E36A IC is 8 kHz, sampling all selected channels simultaneously. Acquired raw samples are sent over the SPI bus, which has a maximum bandwidth of 1024 kHz.
• Press  ＋  ＋  to enter the menu.
• Press  ＋  ＋ number of channels ＋  . The number of channels must be between one and five (voltage of channel A and up to 4 current).
  – If you enter 8 ＋ 1: this corresponds to the voltage of channel A.
  – If you enter 8 ＋ 2: corresponds to channel A voltage ＋ channel A current.
  – If you enter 8 ＋ 3: corresponds to channel A voltage ＋ currents in channels A and B.
  – If you enter 8 ＋ 4: corresponds to channel A voltage ＋ currents in channels A, B and C.
  – If you enter 8 ＋ 5: corresponds to channel A voltage ＋ currents in channels A, B, C and D.
• Wait the desired measuring time.
• Press  ＋  ＋  to stop record.
• From the GTK-term save the plain text file with the hexadecimal samples. The hexa string in the serial port terminal is shown in Fig. 17. Save the generated .txt file locally (into Raspberry Pi SD card) to be processed afterwards by program **P2**.
• Use **P2** (example of application available in [18]) to convert the samples in the time domain (Fig. 18, Fig. 19). The Python code (**P2**) was designed to separate, filter, interpret, and convert the raw samples coming from serial communication into a floating data type. For example, to capture three channels on the RealTerm^4^ serial port terminal, the following steps are taken:
  1. Establish communication between the console and Arduino DUE via the appropriate COM port in the terminal port configuration menu and set the transmission baud rate to 100000 baud (Fig. 13).
  2. Press  (0x1b) ＋  (0x38) ＋ number of channels ＋  (0x0d). Send the ASCII command via console. For example, for three channels (0x33) (Fig. 14).
  3. Save the plain text file containing the hexadecimal samples (tick the appropriate option, Fig. 15).
  4. The samples from all enabled ADC channels are sent in an interleaved sequence: I4, I1, V1, I2, V2 and I3, V3. If any channel is disabled, it is not included in the list, while the sequence of the other channels is maintained (Fig. 16 shows an example of the sample sequence.). Use **P2** (example of application available in [18]) to convert to samples in the time domain.

### 6.3 M90E36A IC internal DSP usage and register RMS values

To access the time base registers of the M90E36A IC and start recording variables to the SD memory, use the serial port communication console and:

• Set the console transmission speed to 115200 bauds in port configuration menu (Fig. 10).
• Press  ＋  ＋  to enter menu.
• Press  ＋  ＋ specific time ＋  . Use specific time in ms between 333 to 6000.
• Press  ＋  ＋  to stop record.

Then, a CSV file will be stored in memory with the following structure (labels) in each row:

‘Day’, ‘Time’, ‘UrmsA(V)’, ‘UrmsB (V)’, ‘UrmsC (V)’, ‘IrmsA (A)’, ‘IrmsB (A)’, ‘IrmsC (A)’, ‘IrmsN (A)’, ‘PmeanA (W)’, ‘PmeanB (W)’, ‘PmeanC (W)’, ‘PmeanT (W)’, ‘QmeanA (VAR)’, ‘QmeanB (VAR)’, ‘QmeanC (VAR)’, ‘SmeanA (VA)’, ‘SmeanB (VA)’, ‘SmeanC (VA)’, ‘PFmeanA’, ‘PFmeanB’, ‘PFmeanC’, ‘THDNUA(%)’, ‘THDNUB(%)’, ‘THDNUC(%)’, ‘THDNIA(%)’, ‘THDNIB(%)’, ‘THDNIC(%)’, ‘Frec.(Hz)’, ‘Temp.(°C)’.

Where, ‘UrmsA (V)’ is the RMS phase voltage, ‘IrmsA (A)’ is the phase current, ‘PmeanA (W)’ is the average active power, ‘QmeanA (VAR)’ is the average reactive power, ‘SmeanA (VA)’ is the average apparent power, ‘PFmeanA (VA)’ is the average power factor, ‘PFmeanA’ is the average power factor, ‘THDNUA(%)’ is the voltage harmonic distortion rate (in percent of fundamental), and ‘THDNIA(%)’ is the current harmonic distortion rate (in percent of fundamental). All of them for the phase associated with channel A as indicated with the last letter. The same quantities are reported for channels B and C following the same logic. ‘Frec.(Hz)’ is the frequency of the signal and ‘Temp. (°C)’ is the ambient temperature.

**Fig. 20:**
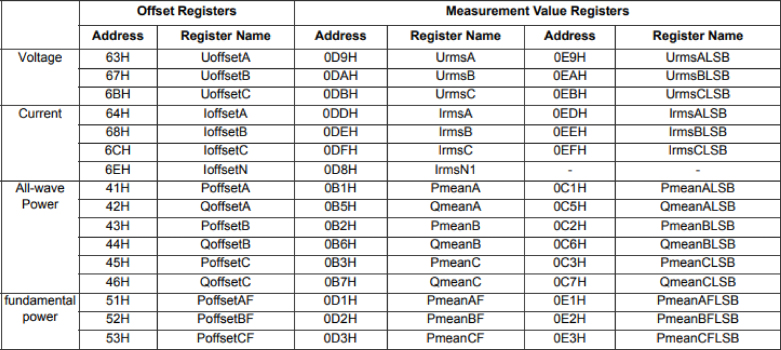
Measurement value registers [15].

### 6.4 Calibration

The M90E32A IC provides a compensated calibration function for voltage, current, and power that reduces the influence of the interference signal on the measurement accuracy. For our application, the voltage and current registers were calibrated. The calibration process requires the use of a test bench in order to produce the required settings in each of the phases (detailed in the next section). Different operating points of the equipment are established for the calibration of the gain and the offset for each channel. The calibration registers are shown in Fig. 20.

**Fig. 21:**
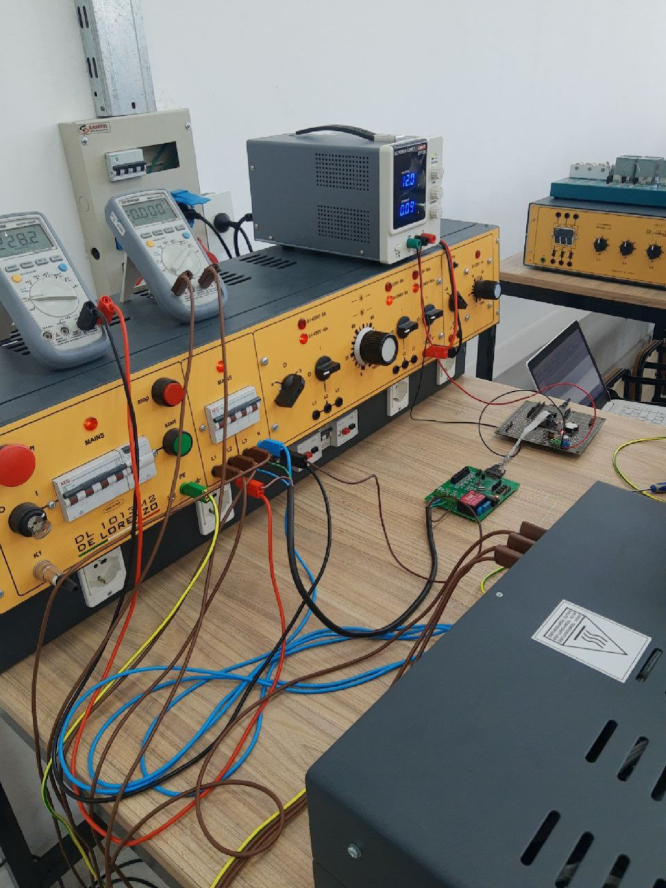
Test bench.

This stage of the project was carried out in the UNAHUR [22] Electrical Engineering Laboratory (Fig. 21), having three-phase voltage generators and resistive loads, so that different power conditions and loads could be emulated, as well as the possibility of carrying out zero- and full-scale calibrations of the variables involved. The calibration was carried out on a test bench composed by:

• De Lorenzo DL 1013 M2 module (configurable three-phase power supply) [23].
• De Lorenzo DL 1017R module (configurable resistive bench) [24].

The measurements were carried out on a three-phase star with neutral circuit.

Offset calibration process: The voltage–current compensation calibration flow is as follows, according to the procedure described in the application note [15]. Each phase power offset calibration should be performed individually. For example, take phase A, the signal source should be set as: $U_{A}=U_{B}=U_{C}=U_{N}$, $I_{A}=0$, and $I_{B}=I_{C}=I_{N}$, where $U_{i},I_{i}$ correspond to the voltage and current in each of the phases i=A,B,C, $U_{N}$ is the nominal voltage and $I_{N}$ is the nominal current.
  To calibrate the system, you must enter the calibration menu sending the  ＋  ＋  command and following the steps that appear on the console. Throughout the procedure (step-by-step cycling through the phases), the different calibration registers of the M90E36A IC are written using the externally measured values at each step of the calibration process. The calibration registers of the M90E36A are shown in Fig. 20.
Gain Calibration Process: For the gain calibration, all three phases are calibrated simultaneously with the currents and voltages at their respective nominal values.
  The calibration method is as follows:
  1. The voltage/current value of the external reference meter^5^ is read, and the same is done for the voltage/current measurement value in the chip records (‘voltage_measurement_value’ and ‘current_measurement_value’).
  2. The system will request the corresponding external readings to update the gain according to the expressions (2), (3), following [15].
    (2) $$new\text{\_}current\text{\_}gain=\frac{reference\text{\_}current\text{\_}value}{current\text{\_}measurement\text{\_}value}\cdot existing\text{\_}current\text{\_}gain$$
    (3) $$new\text{\_}voltage\text{\_}gain=\frac{reference\text{\_}voltage\text{\_}value}{voltage\text{\_}measurement\text{\_}value}\cdot existing\text{\_}voltage\text{\_}gain$$

## 7 Validation and characterization

### 7.1 Communication and transmission verification

**Fig. 22:**
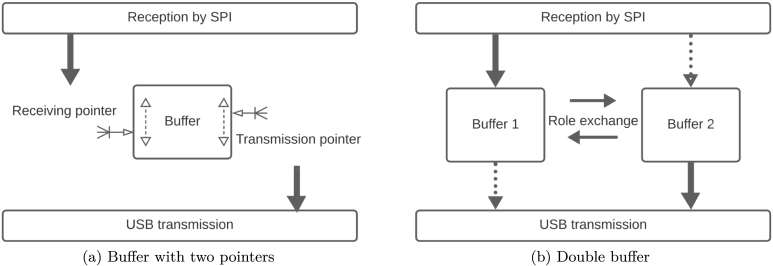
Buffer configurations evaluated.

In the first instance, for DMA operation it was proposed to work with a single buffer of 256 bytes, which had two pointers associated with it (Fig. 22(a)). One of them for writing data, handled exclusively by the interrupt dedicated to SPI communication; and the second for reading, dedicated to data transmission over the USB communication channel. In this way, the SPI interrupt adds data to the buffer using the reception and restarts automatically when it is full (cyclic buffer), while the USB transmission process constantly checks for data to send, thus causing the permanent emptying of the buffer through the transmission pointer.

At a fixed sampling rate of 8 kHz (in DMA mode), using 2 channels and 3 bytes per sample, a transmission rate of 384000 B/s is obtained. The SPI channel has a higher bandwidth capacity and can reach 10 MHz, so there is no problem in transferring data from the M90E36A-DB to the ACQII. The dilemma arises when trying to work with more than two channels in DMA mode. In this case, sequence errors were detected in the transmission from the ACQII to the Raspberry Pi. An attempt was made to improve the transmission by gradually increasing the size of the buffer, but this did not produce the expected result. Trying different buffer sizes did not improve the transmission as sequence errors and missing values were still detected. We therefore had to opt for a double buffer solution.

The solution adopted was to implement a transmission scheme using a double buffer (Fig. 22(b)). Once this scheme is in place, it is obvious that each of them cannot have a single function (reception or transmission), since sometimes they have to receive data via SPI and sometimes they have to transmit this data via USB to be emptied. This implies that each of them has to fulfill a double role, sometimes receiving and sometimes transmitting, while maintaining the absolute integrity of the data and its temporal sequence, since an error in this sequence would change the format of the transmitted data. Flags are therefore included to indicate whether each buffer is being used for reception via SPI or for transmission via USB. In this way, while one buffer is busy receiving data, the other is busy sending data. The roles are then swapped, with sequential integrity ensured by operational flags. As the processor in question (Cortex-M3 in Arduino DUE) operates at a frequency of 84 Mhz, it has been verified that it is far from being stressed by this data management, leaving it time to carry out all the operational tasks required. The processor frequency used is 84 MHz and was considered sufficient as the bandwidth used in the SPI does not exceed 1024 kB/s. As the processor, when working in DMA with the M90E36A IC, only reads the raw data and returns it without any processing; and has a double communication buffer, it was observed that it remained stable and highly reliable over time, not losing any of the bytes read and sent.

### 7.2 Application example

**Fig. 23:**
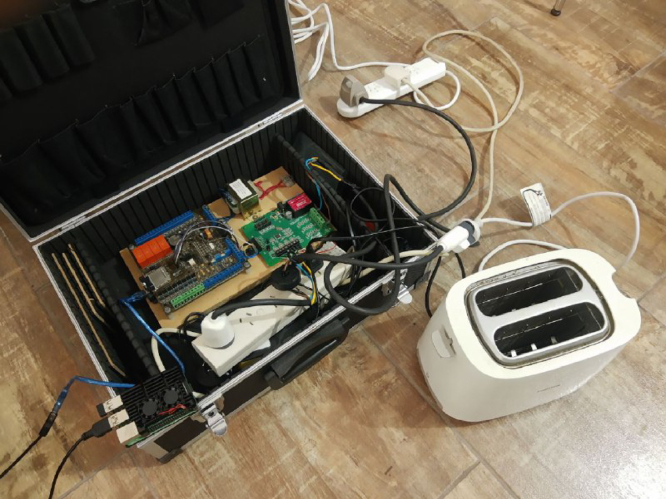
Hardware *ad hoc* application example.

We present a simple example of signal processing developing a specific DSP for energy disaggregation purposes. In this case, we consider the evolution of residual harmonic currents over transient loading periods that could make up a database of specific representative features for each individual appliance. This is an example the kind of features that could be used to develop disaggregation algorithms [25]. We apply the developed hardware to the consumption characteristics of the 700 W domestic toaster shown in Fig. 23.

**Fig. 24:**
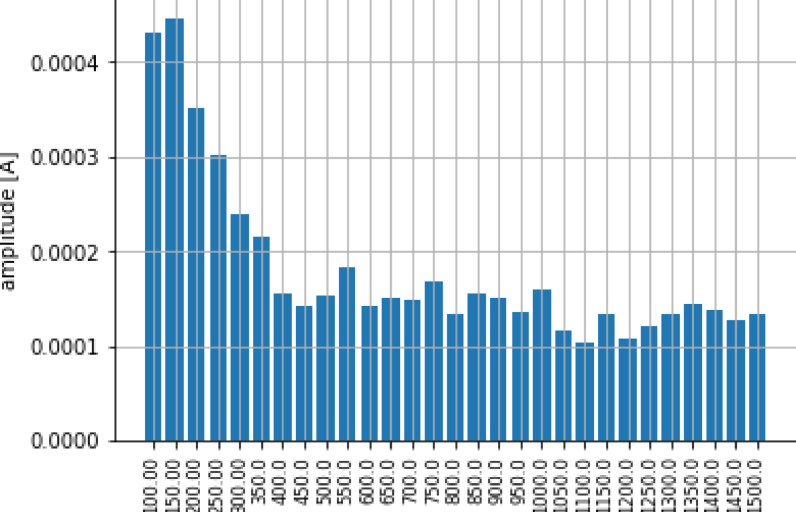
Amplitude of the first 30 harmonics in a sample 1 s window.

**Fig. 25:**
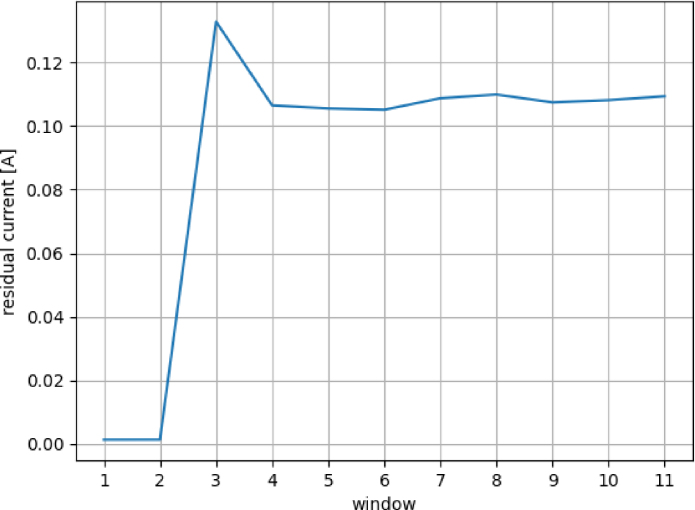
Evolution of harmonic residual current for windows.

The current is sampled at a rate of 8 kHz during the analysis of the transient phenomenon and divided into 1-second windows. For each window, a Discrete Fourier transform (DFT) is computed (e.g. Fig. 24) for characterizing the residual current variability. It can be defined as the square root of the sum of all harmonic components of the current, with the exception of the fundamental one [26], according to

(4) $$I_{residual}=\sqrt{\overset{n}{\underset{h=2}{\sum }}I_{h}^{2}},$$

where $I_{h}$ is the modulus of the Fourier coefficient on the h-mode. In this example, 30 harmonic components were considered (i.e. n=30). The resulting evolution of the residual current of the toaster during a transient event is shown in Fig. 25. There it can be seen that the $I_{residual}$ increases in the first window after it is powered on an then stabilizes in the following windows where the appliance is kept on.

### 7.3 Limitations

• The accuracy of the embedded system depends on the transducers used and the external instruments (standards) used in the calibration procedure.

## Ethics statements

This work did not involve human subjects nor animal experiments.

## CRediT authorship contribution statement

**Maximiliano E. Véliz:** Writing – original draft, Software, Resources, Methodology, Investigation, Formal analysis, Conceptualization. **Gustavo E. Real:** Supervision, Investigation, Formal analysis. **Alejandro D. Otero:** Writing – review & editing, Supervision, Project administration, Methodology, Investigation.

## Declaration of competing interest

The authors declare that they have no known competing financial interests or personal relationships that could have appeared to influence the work reported in this paper.

## Footnotes

1 https://github.com/Jeija/gtkterm.

3 This approximate speed arises from considering the transmission of 24-bit data, with a sampling of 8 kHz through 5 channels.

4 https://realterm.en.lo4d.com.

5 In all cases the external measurement corresponding to the ‘reference_voltage_value’ and ‘reference_current_value’ were obtained with a $3^{\frac{1}{2}}$ digit multimeter.

## Footnotes

2 The full list of commands is available in [19].
